# Racial bias and reproducibility in pulse oximetry among medical and surgical inpatients in general care in the Veterans Health Administration 2013-19: multicenter, retrospective cohort study 

**DOI:** 10.1136/bmj-2021-069775

**Published:** 2022-07-06

**Authors:** Valeria S M Valbuena, Sarah Seelye, Michael W Sjoding, Thomas S Valley, Robert P Dickson, Steven E Gay, Dru Claar, Hallie C Prescott, Theodore J Iwashyna

**Affiliations:** 1 Department of Surgery, University of Michigan, Ann Arbor, MI, USA; 2 Veterans Affairs Center for Clinical Management Research, Ann Arbor, MI, USA; 3 National Clinician Scholars Program, University of Michigan, Ann Arbor, MI, USA; 4 Department of Internal Medicine, University of Michigan, Ann Arbor, MI, USA

## Abstract

**Objectives:**

To evaluate measurement discrepancies by race between pulse oximetry and arterial oxygen saturation (as measured in arterial blood gas) among inpatients not in intensive care.

**Design:**

Multicenter, retrospective cohort study using electronic medical records from general care medical and surgical inpatients.

**Setting:**

Veteran Health Administration, a national and racially diverse integrated health system in the United States, from 2013 to 2019.

**Participants:**

Adult inpatients in general care (medical and surgical), in Veteran Health Administration medical centers.

**Main outcomes measures:**

Occult hypoxemia (defined as arterial blood oxygen saturation (SaO_2_) of <88% despite a pulse oximetry (SpO_2_) reading of ≥92%), and whether rates of occult hypoxemia varied by race and ethnic origin.

**Results:**

A total of 30 039 pairs of SpO_2_-SaO_2_ readings made within 10 minutes of each other were identified during the study. These pairs were predominantly among non-Hispanic white (21 918 (73.0%)) patients; non-Hispanic black patients and Hispanic or Latino patients accounted for 6498 (21.6%) and 1623 (5.4%) pairs in the sample, respectively. Among SpO_2_ values greater or equal to 92%, unadjusted probabilities of occult hypoxemia were 15.6% (95% confidence interval 15.0% to 16.1%) in white patients, 19.6% (18.6% to 20.6%) in black patients (P<0.001 *v* white patients, with similar P values in adjusted models), and 16.2% (14.4% to 18.1%) in Hispanic or Latino patients (P=0.53 *v* white patients, P<0.05 in adjusted models). This result was consistent in SpO_2_-SaO_2_ pairs restricted to occur within 5 minutes and 2 minutes. In white patients, an initial SpO_2_-SaO_2_ pair with little difference in saturation was associated with a 2.7% (95% confidence interval −0.1% to 5.5%) probability of SaO_2_ <88% on a later paired SpO_2_-SaO_2_ reading showing an SpO_2_ of 92%, but black patients had a higher probability (12.9% (−3.3% to 29.0%)).

**Conclusions:**

In general care inpatient settings across the Veterans Health Administration where paired readings of arterial blood gas (SaO_2_) and pulse oximetry (SpO_2_) were obtained, black patients had higher odds than white patients of having occult hypoxemia noted on arterial blood gas but not detected by pulse oximetry. This difference could limit access to supplemental oxygen and other more intensive support and treatments for black patients.

## Introduction

Pulse oximetry is a ubiquitous technology with applications in both ambulatory and inpatient settings. Despite its widespread use, variation in device accuracy by patient race in critically ill patients has been reported as early as 1990.^1^ ^2^ ^3^ Recent investigations have documented differential accuracy in pulse oximetry measurement between black and white patients in intensive care units and in critically ill patients with respiratory failure, with black patients having a higher prevalence of occult hypoxemia than white patients.^4^ ^5^ ^6^ ^7^ ^8^ Occult hypoxemia is defined as having a low saturation of arterial blood gas (that is, SaO_2_ <88%) despite seemingly normal pulse oximetry (that is, SpO_2_ ≥92%). Among critically ill patients, patients with occult hypoxemia detected by arterial blood gas but missed by pulse oximetry have recently been shown to have worse clinical outcomes by detailed analyses—including higher mortality and greater incidence of organ failure—as might be expected given the central role of oxygen delivery in healthy cellular functioning.^6^ ^9^

Policy and regulatory interest over the technology has increased,^10^ ^11^ ^12^ and independent expert bodies have called for additional research in the subject.^13^ ^14^ However, several pressing gaps in the research limit the bedside application of existing data. First, most patients in hospital are not critically ill, and it is unclear whether pulse oximetry bias observed in critically ill patients can be generalized to less acute settings. Second, many laboratory studies have focused on the accuracy of pulse oximeter readings with SpO_2_ <85%.^2^ ^3^ ^15^ If concerns about occult hypoxemia and pulse oximeter bias do not apply to less ill patients with SpO_2_ readings of ≥92%, acting on existing data could lead to unnecessary invasive measurement of arterial blood gases or unnecessary expenditures on new pulse oximeters. Finally, while SpO_2_-SaO_2_ discrepancies are more common in black patients than in white patients, the stability of such discrepancies within an individual patient is unclear. Thus, if a clinician has documented such a discrepancy (or its absence) and the same patient later shows signs or symptoms compatible with arterial hypoxemia, the next step is unclear: whether to repeat an arterial blood gas measurement, or to assume the same direction and magnitude of the SpO_2_-SaO_2_ discrepancy as recently documented.

We sought to correct these gaps in this study of medical and surgical inpatients in general care in the Veterans Health Administration, a large and diverse health system serving veterans across the US. We hypothesized that black patients in general care (that is, not in intensive care) would have an increased frequency of occult hypoxemia compared with white inpatients in general care. We also hypothesized that a blood gas measurement showing the absence of occult hypoxemia would be associated with a low probability of occult hypoxemia on subsequent arterial blood gases, and that this probability would not vary by race. As in past work, we analyzed spontaneously recorded SpO_2_-SaO_2_ measurements collected during routine care from inpatients in the Veterans Health Administration from 2013 to 2019.^16^

## Methods

### Study setting and data source

The nationwide Veterans Health Administration provides comprehensive inpatient and outpatient medical care to US veterans in over 100 hospitals. During the study period, Veterans Health Administration hospitals used one electronic health record system (Computerized Patient Record System), which archived data to a central repository (Corporate Data Warehouse). During the study (2013-19), we extracted SpO_2_ and SaO_2_ data from the Corporate Data Warehouse for all hospital stays in acute medical and surgical care in the Veterans Affairs Patient Database (VAPD). 16 The VAPD, described previously,^17^ is a retrospective cohort study that includes clinical data on all acute inpatient admissions to Veterans Health Administration hospitals. SpO_2_ values and time of pulse oximetry measurement on general care floors are generally entered into the electronic health record by the clinician measuring them; SaO_2_ values of arterial blood gas and timing of the blood draw come directly from laboratory reporting systems.

### Primary outcome—occult hypoxemia

We defined occult hypoxemia as a low saturation of arterial oxygen (SaO_2_ <88%) with a pulse oximetry reading of SpO_2_ ≥92% recorded at the same time. This range of SpO_2_ was selected because clinicians would probably not increase oxygen levels in response to SpO_2_ ≥92%.^18^ For all analyses, SpO_2_ was included as a continuous variable. All pairs of SpO_2_ and SaO_2_ values that were measured within 10 minutes for patients in the VAPD were identified. We excluded SaO_2_ measurements that were labeled as temperature corrected or calculated, and information on whether the SaO_2_ reading was measured by co-oximetry was not available. To remove critically ill patients and isolate a sample consisting of patients in general care for these analyses, we excluded pairs of readings that were measured on days when patients were in intensive care or transferred in or out of intensive care. For the same reason, we also excluded pairs of SpO_2_-SaO_2_ readings from the rare days when more than two blood gases were drawn. We removed SaO_2_ and SpO_2_ values lower than 70% to reduce the possibility of mislabeling a venous blood gas as an arterial blood gas or data entry error, because such values seemed unlikely to be accurate in patients who were not critically ill.

### Race and ethnic origin

The VAPD includes patient demographic data on race and ethnic origin. Six categories by race and ethnic origin were used: non-Hispanic black, non-Hispanic white, Hispanic or Latino, Asian or Pacific Islander, American Indian, and other. These categories were assigned through a variety of administrative processes in the Veterans Health Administration, including self-report. In order to be generated, valid VAPD records require data on core identifiers (that is, age, race and ethnic origin, and sex); therefore, these data were complete for all analyses. For race and ethnic origin, adjusted models that predicted occult hypoxemia included non-Hispanic black (referred to in this article as “black”), non-Hispanic white (“white”), and Hispanic or Latino (“Hispanic”), consistent with previous research on racial and ethnic differences of occult hypoxemia being most reproducible between those groups.^5^

### Statistical analyses

Analyses included all patient days with one or two pairs of SpO_2_-SaO_2_ reading on that day; χ^2^ and independent t tests were conducted to compare patient characteristics by race and ethnic origin. We fit a multivariable logistic regression model to predict the odds of occult hypoxemia (SaO_2_ <88% for patients with SpO_2_ ≥92%). Models were adjusted for patient level characteristics that included black race (*v* white race), age, sex, patient comorbidities (appendix 1), supplemental oxygen (as a continuous variable), and diagnoses on admission (including indicators for the 20 most common diagnosis categories within the Healthcare Cost and Utilization Project Clinical Classification Software).^19^ All cause, all location mortality at 30 days from admission was calculated using the Veterans Affairs Vital Status Files. We included a quadratic term for SpO_2_ readings to account for non-linearity in the difference between SpO_2_ and SaO_2_ values, and we included an interaction term for black race and SpO_2_ readings to test the hypothesis that the relation between race and occult hypoxemia is different across values of SpO_2_. Standard errors robust to clusters were used to account for clustering at the patient level.

Because of the complex non-linearities and interactions in the model, we did not report the specific coefficients for race. Instead, we reported predictive margins and average marginal effects of occult hypoxemia for each category of race included in the model (that is, average marginal effects for black patients *v* white patients, and separately for Hispanic patients *v* white patients).

We estimated the potential population level burden of occult hypoxemia among general care inpatients in VA hospitals across the system under the simple assumption that the probability of occult hypoxemia was the same in SpO_2_ readings not paired to an SaO_2_ reading as the probability in SpO_2_ values recorded in paired readings, after adjusting for all the covariates listed above. We applied the predictive margins of occult hypoxemia by race to the full sample of all pulse oximetry readings (for patients not in intensive care) with SpO_2_ values ≥92%. The number of predicted instances of occult hypoxemia, and not unique hospital admissions or unique patients, is rounded to the nearest thousand to emphasize to readers that this number is an estimate.

We calculated bias (mean difference of the SpO_2_ value minus the SaO_2_ value), precision (standard deviation of the differences), limits of agreement (bias ±1.96×standard deviation), and accuracy (root mean square error (ARMS) is the square root of the sum of squared bias and squared precision) by race and ethnic origin for the entire dataset according to previously described methods.^20^ ^21^ ^22^ Lower values for root mean square error indicate a higher degree of correlation between the two measurement modalities evaluated (that is, SaO_2_ and SpO_2_). A root mean square error of ≤2-3% is required by the US Food and Drug Administration (FDA) for initial approval pulse oximetry devices.^20^ ^21^ ^22^ ^23^

Data management and analyses were performed using SAS (SAS Institute, Cary, NC) and Stata/MP version 16.1 (StataCorp, College Station, TX). The analytic code is included in appendix 2, and is available through GitHub. This study followed the STROBE statement (STrengthening the Reporting of OBservational studies in Epidemiology) guidelines^24^ and was approved by the Veterans Health Administration’s institutional review board as part of IRB #1597241 Long term Consequences for Veterans with Sepsis.

### Patient and public involvement

No members of the public were formally involved in the conception of this study, but our interest in the phenomenon of pulse oximetry measurement bias stems from caring for patients from different racial and ethnic backgrounds during the covid-19 pandemic.

## Results

### Patient characteristics

**Fig 1:**
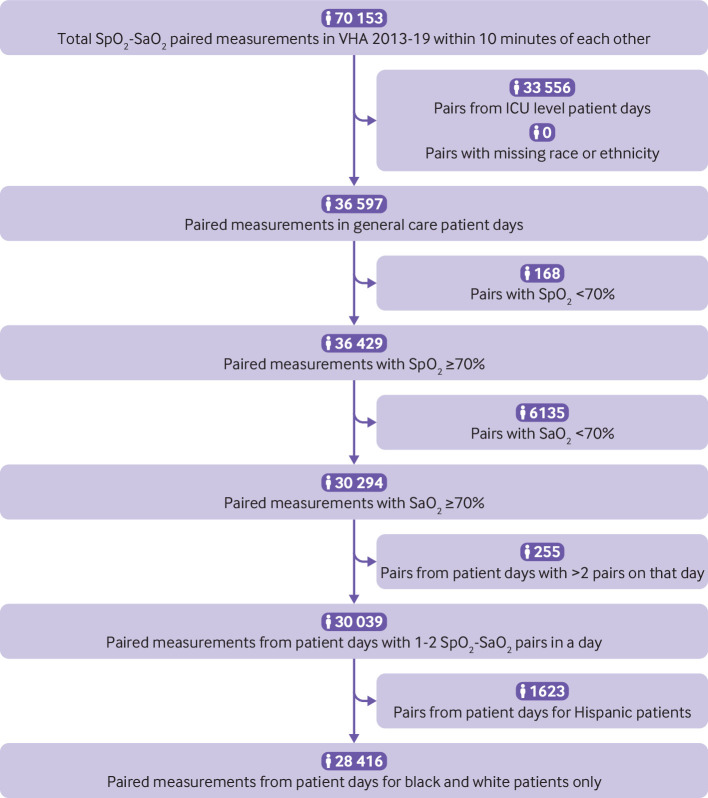
Cohort flow diagram. SaO_2_=arterial blood gas reading; SpO_2_=pulse oximetry reading; VHA=Veterans Health Administration; ICU=intensive care unit; black=non-Hispanic black; white=non-Hispanic white; Hispanic=Hispanic or Latino

We identified 70 153 inpatient pairs of SpO_2_-SaO_2_ readings recorded within 10 minutes of each other during the study period. From these, 33 556 SpO_2_-SaO_2_ pairs were excluded, representing patients who received intensive care at any point on that calendar day. We also excluded 204 pairs from patient days with more than two pairs of readings in one day. The cohort included 30 039 SpO_2_-SaO_2_ pairs (fig 1 and appendix 3), and the analyses had no missing data.

Characteristics of included pairs of SpO_2_-SaO_2_ readings are presented in table 1 (and appendix 4). When the pair of SpO_2_-SaO_2_ readings were recorded, patients were receiving minimal oxygen supplementation (eg, white patients received a mean of 0.9 L/min (standard deviation 2.5)). SaO_2_ readings were slightly more common on days when an SpO_2_ reading was recorded for white patients than for black patients (1.7 *v* 1.5 SaO_2_ readings per 1000 SpO_2_ monitored patient days, odds ratio 0.886 95% confidence interval 0.862 to 0.912)), but if an SaO_2_ reading was available on an SpO_2_ monitored day, no substantial difference by race was seen in whether that reading was close enough in time to be in a pair with a recorded SpO_2_ reading (0.963, 0.935 to 0.991).

**Table 1:** Characteristics of SpO_2_-SaO_2_ pairs recorded in study population, by race and ethnic origin

Characteristics	Non-Hispanic white	Non-Hispanic black	Hispanic/ Latino	P value of difference	
				White ***v*** black	White ***v*** Hispanic
**Paired SpO_2_-SaO_2_ readings**					
Total No	21 918	6498	1623	—	—
Median (IQR) pulse oximetry (SpO_2_, %)	95 (93-97)	97 (94-99)	97 (94-99)	<0.001	<0.001
Median (IQR) arterial oxygen saturation (SaO_2_, %)	94 (89.9-96.6)	94.3 (89-97.1)	95 (90.2-98)	0.31	<0.001
Supplemental oxygen (L/min)					
Median (IQR)	0	0	0	<0.001	0.14
Mean (SD)	0.9 (2.5)	0.6 (2.0)	0.8 (2.7)	<0.001	0.14
Potential occult hypoxemia					
All SpO_2_ (92-100%)	18 157 (82.8)	5852 (90.1)	1466 (90.3)	<0.001	<0.001
SaO_2_ <88% if SpO_2_ is 92-100%	2823 (15.6)	1144 (19.6)	237 (16.2)	<0.001	0.53
**Patient day characteristics**					
Total No	20 822	6190	1519		
Median (IQR) age (years)	69 (64-77)	66 (60-72)	68 (62-76)	<0.001	<0.001
Male sex (No (%))	20 099 (96.5)	5852 (94.5)	1479 (97.4)	<0.001	0.082
Primary diagnoses (No (%))					
Chronic obstructive pulmonary disease	2984 (14.3)	681 (11.0)	97 (6.4)	<0.001	<0.001
Respiratory failure	2766 (13.3)	633 (10.2)	162 (10.7)	<0.001	0.003
Septicemia	1622 (7.8)	466 (7.5)	118 (7.8)	0.50	0.98
Pneumonia	1608 (7.72)	291 (4.7)	59 (3.9)	<0.001	<0.001
Congestive heart failure	1336 (6.4)	464 (7.5)	84 (5.5)	0.003	0.17
Coronary atherosclerosis	391 (1.9)	108 (1.7)	184 (12.1)	0.50	<0.001
Diabetes with complication	309 (1.5)	204 (3.3)	30 (2.0)	<0.001	0.13
Cardiac dysrhythmia	361 (1.7)	94 (1.5)	18 (1.2)	0.25	0.11
Renal failure	316 (1.5)	115 (1.9)	23 (1.5)	0.06	0.99
Acute myocardial infarction	223 (1.1)	58 (0.9)	71 (4.7)	0.36	<0.001
Other	8906 (42.8)	3076 (49.7)	673 (44.3)	<0.001	0.24
Comorbidities* (No (%))					
Congestive heart failure	7304 (35.1)	2202 (35.6)	486 (32.0)	0.47	0.02
Neurological disease	2436 (11.7)	896 (14.5)	175 (11.5)	<0.001	0.834
Chronic pulmonary disease	11 911 (57.2)	2642 (42.7)	495 (32.6)	<0.001	<0.001
Liver disease	1746 (8.4)	694 (11.2)	213 (14.0)	<0.001	<0.001
Diabetes without complication	5099 (24.5)	1732 (28.0)	448 (29.5)	<0.001	<0.001
Diabetes with complication	3480 (16.7)	1171 (18.9)	359 (23.6)	<0.001	<0.001
Non-metastatic cancer	2176 (10.5)	711 (11.5)	146 (9.6)	0.02	0.30
Metastatic cancer	782 (3.8)	266 (4.3)	38 (2.5)	0.05	0.01
Renal disease	4935 (23.7)	2004 (32.4)	447 (29.4)	<0.001	<0.001
Median (IQR) length of hospital stay (days)	6 (4-10)	6 (3-11)	9 (4-17)	<0.001	<0.001
Mortality (No (%))					
In hospital	1118 (5.4)	350 (5.7)	91 (6.0)	0.39	0.30
At 30 days	2240 (10.8)	553 (8.9)	142 (9.4)	<0.001	0.086

SaO_2_=arterial blood gas reading; SpO_2_=pulse oximetry reading; IQR=interquartile range; SD=standard deviation.

### Probability of occult hypoxemia

**Fig 2:**
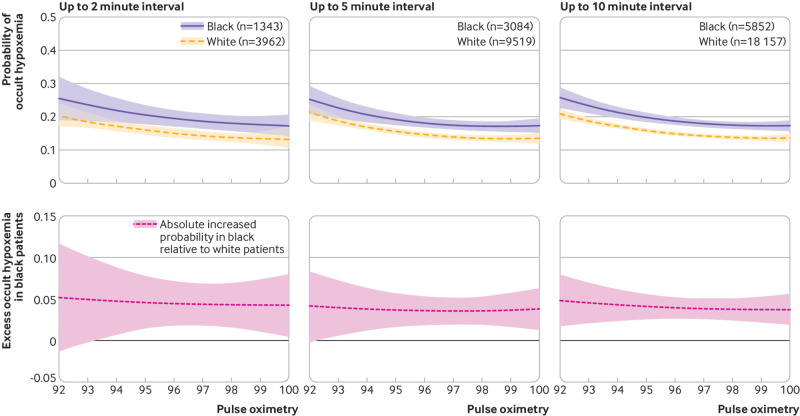
Adjusted rate differences in probability of occult hypoxemia (arterial oxygen saturation SaO_2_ <88% when pulse oximetry SpO_2_ ≥92%) in study population by pulse oximetry reading and by race, from logistic regression model. Model adjusts for age, male sex, comorbidities, and diagnoses, and run only for pulse oximetry (SpO_2_) ≥92% and allowing for non-linear interactions between race and pulse oximetry. Top row of graphs shows estimated predictive margins by race; bottom row of graphs shows the differences between the groups; shaded areas are 95% confidence intervals. Inclusion criteria within columns of graphs is the maximum difference between the time stamp on SaO_2_ collection and the recorded SpO_2_ time. Moving graphs from left to right, analyses included 5305 SpO_2_-SaO_2_ pairs with SpO_2_ readings of ≥92% measured up to 2 minutes apart (median time difference 1.0 minute (interquartile range 0.2-1.5)); 12 603 pairs measured up to 5 minutes apart (2.6 minutes (1.0-4.0)); and 24 009 pairs measured up to 10 minutes apart (5.0 minutes (2.4-7.7)); these numbers of pairs are lower than all possible SpO_2_-SaO_2_ pairs because of restricting SpO_2_ readings to those 92% and over

The number of SpO_2_-SaO_2_ pairs by race and ethnic origin at the SpO_2_ range of 89-100% are summarized in appendix 3. Among all values of SpO_2_ ≥92%, unadjusted probabilities of occult hypoxemia (where SaO_2_ values are <88%) were 15.6% (95% confidence interval 15.0% to 16.1%) for 2823 pairs in white patients, and 19.6% (18.6% to 20.6%) for 1144 pairs in black patients (P<0.001 *v* white; table 1). After adjusting for age, male sex, comorbidities, and diagnoses, and allowing for non-linear interactions between race/ethnic origin and pulse oximetry, the absolute adjusted probability of occult hypoxemia was 4.0% (2.7% to 5.3%; P<0.001) higher in black patients than in white patients (fig 2).

White patients faced less measurement bias in pulse oximetry than black patients (2.3 *v* 4.0), and pulse oximeters had better precision in white patients than in black patients (6.6 *v* 7.3, lower is better in this definition of precision), when the paired SaO_2_ values were used as the gold standard across the entire range of SpO_2_ values in the data (appendix 5).

This estimate of racial differences in occult hypoxemia was not sensitive to time between SpO_2_-SaO_2_ pairs—whether it was calculated for pairs no more than 10 minutes apart (median time difference 5.0 minutes (interquartile range 2.4-7.7) for 24 009 pairs, absolute adjusted increased probability of occult hypoxemia 4.0% (95% confidence interval 2.7% to 5.3%; P<0.001)), for pairs no more than 5 minutes apart (2.6 minutes (1.0-4.0) for 12 603 pairs, 3.7% (2.0% to 5.5%; P<0.001)), or for pairs no more than 2 minutes apart (1.0 minute (0.2-1.5) for 5305 pairs, 4.6% (95% confidence interval 1.9% to 7.2%; P=0.001); fig 2).

In total, we observed 54 048 788 SpO_2_ readings of 92-100% for black and white inpatients in the study. If occult hypoxemia occurred at the same rate as occult hypoxemia in the SpO_2_-SaO_2_ pairs analyzed in the study, then an estimated 573 000 additional instances of occult hypoxemia would have occurred in black patients during the study period and would have been detected if pulse oximeters performed as well in black patients as in white patients.

### Patient level divergence between first and second SpO_2_-SaO_2_ pairs

**Fig 3:**
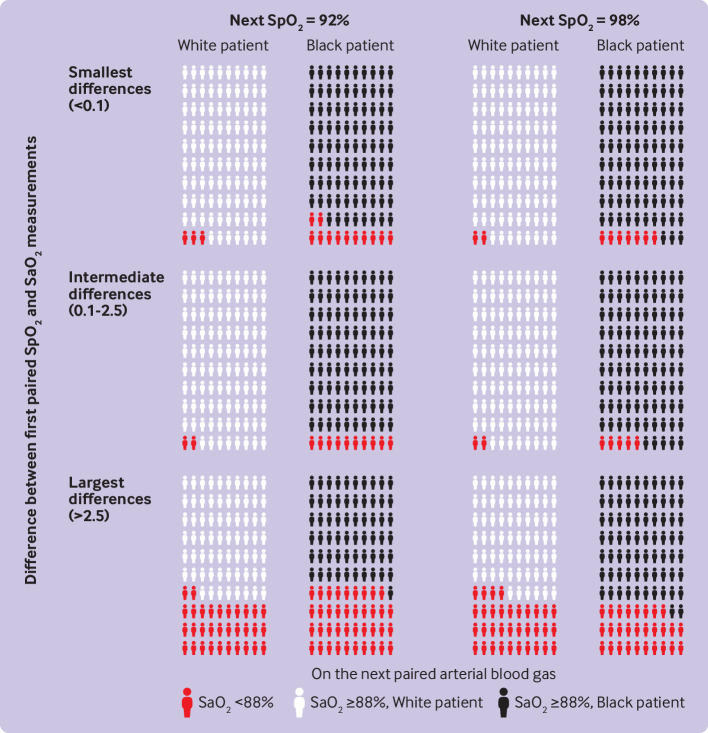
Probability of occult hypoxemia (arterial oxygen saturation SaO_2_ <88% when pulse oximetry SpO_2_ ≥92%) on second paired SpO_2_-SaO_2_ measurements, by race. Probability is based on the SpO_2_-SaO_2_ difference from a first pair of readings measured earlier that same day, race of the patient, and pulse oximetry reading at the time of measurement of the second pair of SpO_2_-SaO_2_ readings. Adjusted probabilities were calculated from a regression stratified by race, and presented using www.iconarray.com visualization recommendations. SpO_2_-SaO_2_ differences were divided into groups by tertiles from the first reading of the day; these groups ranged from having the lowest SpO_2_-SaO_2_ difference (SaO_2_ 0.1 percentage points lower than or any amount higher than SpO_2_) to having the largest SpO_2_-SaO_2_ difference (SaO_2_2 at least 2.5 percentage points lower than SpO_2_). Probabilities of occult hypoxemia on the second pair of SpO_2_-SaO_2_ readings depended on the magnitude of the difference on the first pair, and as a function of the SpO_2_ at the second pair and the patient’s race

We recorded a total of 3016 patient days (for inpatients in general care) that had two pairs of SpO_2_-SaO_2_ measurements recorded on the same day. We aimed to determine whether the difference within SpO_2_-SaO_2_ readings from the first pair was associated with the odds of occult hypoxemia in the second pair. In figure 3, we divided SpO_2_-SaO_2_ differences from the first reading of the day into equal groups by tertiles. These groups ranged from having the lowest SpO_2_-SaO_2_ difference (SaO_2_ reading is 0.1 percentage points lower than or any amount higher than SpO_2_ reading) to having the largest SpO_2_-SaO_2_ difference (SaO_2_ reading is at least 2.5 percentage points lower than SpO_2_ reading).

For example, probabilities of occult hypoxemia for white patients with an SpO_2_ of 92% in the second pair of readings were 2.7% (95% confidence interval −0.1% to 5.5%) if the first pair was in the group with the smallest SpO_2_-SaO_2_ difference, 2.4% (−0.1% to 4.8%) if the first pair had an SpO_2_-SaO_2_ difference in the intermediate group, but 32.0% (15.2% to 48.8%) if the first pair in the group with the largest SpO_2_-SaO_2_ difference. Similar patterns in white patients were seen with SpO_2_ readings of 98% in the second pair. Probabilities of occult hypoxemia of 2.4% (0.4% to 4.5%) and 2.1% (0.3% to 3.9%) were recorded in the groups with the smallest and intermediate SpO_2_-SaO_2_ differences, respectively, but 33.8% (26.1% to 41.50%) if the SpO_2_-SaO_2_ difference was in the highest group (fig 3). 

The probabilities of occult hypoxemia on the second SpO_2_-SaO_2_ pair were higher for black patients than for white patients in the groups with the smallest and intermediate differences—for example, black patients with the lowest SpO_2_-SaO_2_ differences in the first pair and an SpO_2_ reading of 92% in the second pair still had a 12.9% (95% confidence interval −3.3% to 29.0%) probability of hypoxemia. The probability of occult hypoxemia in black patients with the largest SpO_2_-SaO_2_ differences on the first pair was as high as in white patients if the SpO_2_ value in the second pair was 92% (39.6% (11.5% to 67.7%), and even when the SpO_2_ value was 98% (28.4% (18.5% to 38.2%); fig 3).

To put it another way, the odds of occult hypoxemia on the second pair of SpO_2_-SaO_2_ readings varied depending on magnitude of the difference in the first pair, SpO_2_ value in the second pair, and patient race. For example, for white patients who did not show occult hypoxemia in the first pair of SpO_2_-SaO_2_ readings of a given day, occult hypoxemia was present in only 2.5% of the second pair of SpO_2_-SaO_2_ measurements. Among black patients with two pairs of SpO_2_-SaO_2_ readings in a given day and with no occult hypoxemia shown in the first pair, occult hypoxemia was present in 6.5% of the second pair of SpO_2_-SaO_2_ measurements. While the two SpO_2_-SaO_2_ pairs had, on average, the same difference between SaO_2_ and SpO_2_ (mean 0.05 for white patients and 0.15 for black patients), the standard deviation in differences between the pairs was 4.1 for white patients and 4.9 for black patients, indicating that the possibility of wide discrepancy was more common in black patients.

### Post hoc, hypothesis generating, and sensitivity analyses

We also analyzed the results of 1623 pairs of SpO_2_-SaO_2_ measurements from patients identified as Hispanic (or Latino). Among all pairs with SpO_2_ readings of ≥92%, the unadjusted probability of occult hypoxemia (SaO_2_ <88%) was 16.2% (95% confidence interval 14.4% to 18.1%) in these patients (P=0.53 *v* white patients; table 1). After adjusting for age, male sex, comorbidities, and diagnoses, and allowing for non-linear interactions between race and ethnic origin and pulse oximetry, the absolute adjusted rate of occult hypoxemia was 2.5% (95% confidence interval 0.04% to 5.0%; P<0.05) higher in Hispanic patients than in white patients (appendix 6). White patients had less measurement bias in pulse oximetry than Hispanic patients (2.3 *v* 3.3), and pulse oximeters had similar precision in Hispanic patients (6.6 *v* 6.7), when the paired SaO_2_ was used as the gold standard across the entire range of SpO_2_ in the data (appendix 5). Because of the small sample size, we did not further analyze this subgroup of patients.

Focusing on differences between black and white patients, our primary analyses in figure 2 used data for SpO_2_ readings of 92-100%. Including all readings did not meaningfully change the results (appendix 7). Controlling for clustering within hospitals as a random effect did not meaningfully change the results (appendix 8). Analyses testing for a difference in the observed race differences in measurement bias in surgical and non-surgical patients yielded inconsistent results that were sensitive to subtle parameterization choices in the small (<10% of sample) population of patients in surgical general care with SpO_2_-SaO_2_ pairs, but did not change the estimated differences meaningfully in the large majority of patients who were not in surgical care (appendix 9).

## Discussion

### Principal findings

This study of inpatients in general care in the Veterans Health Administration indicates a significant difference in the ability of pulse oximetry to detect clinically relevant hypoxemia in patients of different races. Black patients were found to have more occult hypoxemia than white patients. Pulse oximetry readings had greater bias and worse precision among black inpatients in general care than among white inpatients in general care. This greater bias and worse precision meant that on receiving a recent and well correlated pair of SpO_2_-SaO_2_ readings, white patients could have some reassurance that a later normal SpO_2_ reading was unlikely to be associated with a SaO_2_ reading of <88%; however, less reassurance was available for black patients. The overall prevalence of occult hypoxemia could be considerable and racially biased.

### Comparison with other studies

Our findings accord with the most recent data on racial bias in pulse oximetry focused on more ill patients, often in intensive care.^4^ ^5^ ^6^ ^9^ More recently, an investigation in preterm infants of different races showed a modest but consistent difference in SpO_2_ measurement bias between black and white patients, where the odds of occult hypoxemia were higher in black preterm infants,^8^ as did another study in older children.^25^ However, in a study of 19 black adult patients, researchers reported no significant difference in accuracy by race, although this study might not have been appropriately powered to detect clinically important differences between groups.^7^ In controlled laboratory studies with sometimes modest numbers of largely healthy participants, results have been variable but often show racial inequalities.^2^ ^3^ ^26^ ^27^ ^28^ ^29^

Our results add to the literature by showing that the concerns about worse bias and noise (eg, measurement imprecision) in black patients being monitored with pulse oximetry are also relevant to patients in general care who often receive no or little supplemental oxygen. They also suggest the possibility that this differential pulse oximeter performance affects Hispanic patients, although those results need additional confirmation. Two independent groups have recently shown that arterial hypoxemia undetected by pulse oximetry is associated with worse clinical outcomes.^6^ ^9^

Our study looks at concerns about possible differential bias being introduced because of the potential interval between SpO_2_ and SaO_2_ readings. These data indicate that excess occult hypoxemia is present in black patients relative to white patients, regardless of whether the SpO_2_-SaO_2_ pairs are measured within 2, 5, or 10 minutes apart, suggesting that time between measurements is not a driver of these results—consistent with other recently published work.^9^

Errors in pulse oximeters could be due to a combination of systematic error or bias, which is reproducible across measurements, as well as random error or noise.^30^ Because pulse oximeter error is due to a combination of both processes, the magnitude of pulse oximeter error might not be the same each time a reading is taken. We empirically show that these errors could result in clinically meaningful differences in the interpretation of pulse oximetry across racial groups. In patients with two pairs of SpO_2_-SaO_2_ readings measured on the same day, a well aligned SpO_2_-SaO_2_ pair for white patients was associated with low levels of occult hypoxemia on subsequent pairs; such concordance might be reassuring in many clinical scenarios. This concordance was less true for black patients, and these differences should be considered in deciding whether to obtain an arterial blood gas reading in appropriate clinical situations until non-racially biased pulse oximeters are in use.

### Strengths and limitations

This study had several limitations. Arterial blood gases are measured less frequently than pulse oximetry. If clinicians are more likely to obtain an arterial blood gas when they have concerns about the accuracy of pulse oximetry, then the probabilities of occult hypoxemia noted in our study would overestimate the frequency with which an SpO_2_ reading of ≥92% is truly associated with an SaO_2_ reading of <88%. However, for this non-random arterial blood sampling to explain the apparent differences between racial groups, it would be necessary that clinicians are better at making the bedside diagnosis of occult hypoxemia in black patients than in white patients—and, at the same time, that they order confirmatory tests less often but more accurately in black patients. We are unaware of data that support such an alternative mechanism.

Skin color is not consistently recorded as part of the medical record, so we used race as a surrogate, which might not fully reflect the skin tone diversity within each patient group or other differences that might contribute to pulse oximetry bias. Data on Hispanic patients suggest a similar problem, but the smaller sample size resulted in more imprecise estimates; these data should be considered as hypothesis generating unless replicated. Likewise, the potential instability of estimates of racial differentials in surgical patients warrants additional exploration, although should be taken in the light of other published findings.^9^ Although the age distribution of these data are not unusual for patients in hospital,^31^ most of the patients in this study were male and elderly, potentially limiting the generalizability of our findings; potential interactions of sex and race are not excluded by our study.

An additional limitation was that nearly all patients had a military service history. No oximeter brand information was available for this study. However, most commercially available oximeters use similar technology.^32^ We have no information about the quality of the pulse oximetry signal in these patients—but we know that clinical staff (often including nurses or respiratory therapists) recorded it in the medical record as a valid reading. The estimate of more than half a million possible excess instances of occult hypoxemia among black patients in 2013-19 is based on simplifying assumptions as explained above; to the extent that these assumptions are not true, the estimate will be inflated or deflated.

### Policy implications and conclusions

Black patients under the inpatient care of the US Veterans Administration had excess episodes of undetected hypoxemia in 2013-19 compared with white patients. Other scientists have reported increased morbidity and mortality associated with occult hypoxemia.^6^ ^9^ Much like the NHS in the UK, the Veterans Health Administration is the largest integrated healthcare system in the US, and it has historically set a standard of quality improvement, care coordination, and innovation. Large integrated health systems such as the Veterans Health Administration and NHS could have a role to purchase and use only pulse oximeters proven to provide equivalent accuracy in black patients rather than devices of unproven equity. 33 34

**What is already known on this topic**

Pulse oximetry (SpO_2_) is a method used to non-invasively measure arterial oxygen saturation (SaO_2_); the accepted most accurate method is by invasive arterial blood gas
Patients in intensive care with occult hypoxemia (defined as SaO_2_ <88% despite an SpO_2_ ≥92%) experience worse organ dysfunction and mortality rates in hospital than those without occult hypoxemia
Differential and racially biased performance of pulse oximetry has been reported in predominantly critically ill populations, but little is known about the prevalence and population burden of these differences for inpatients in general care or in large nationwide health systems

**What this study adds**

For individuals in general (medical and surgical) care in hospital, occult hypoxemia was more common for black patients than for white patients; differences in occult hypoxemia by race from real world data did not change according to differences between the recording of the SpO_2_ and SaO_2_ readings, up to at least 10 minutes apart
White patients not showing occult hypoxemia on a first pair of SpO_2_-SaO_2_ readings in a day were unlikely to have it on a second pair of SpO_2_-SaO_2_ readings on the same day; black patients had less consistency across different pairs of SpO_2_-SaO_2_ measurements on the same day
These results suggest that if a recent SaO_2_ reading does not show occult hypoxemia in a black patient, an elevated index of suspicion might be warranted in black patients with compatible signs and symptoms until unbiased pulse oximeters are routinely available

## Data Availability

Technical appendix and statistical code are available in appendix 2 and on GitHub. The dataset used for this analysis is not publicly available.

## Web extra

Extra material supplied by authors

Web appendix: Supplementary material

Web appendix: Analytic code

## Notes

Contributors: VSMV and SS contributed equally to this work. VSMV, SS, and TJI had full access to all the data in the study and take responsibility for the integrity of the data and the accuracy of the data analysis. TJI provided full supervision of all design, analysis, interpretation, and writing stages of the manuscript. MWS, TSV, RPD, DC, and HCP contributed substantially to the study design, data analysis and interpretation, and the writing of the manuscript. TJI is the study guarantor. The corresponding author attests that all listed authors meet authorship criteria and that no others meeting the criteria have been omitted.

The lead author (the manuscript’s guarantor) affirms that the manuscript is an honest, accurate, and transparent account of the study being reported; that no important aspects of the study have been omitted; and that any discrepancies from the study as planned (and, if relevant, registered) have been explained.

Dissemination to participants and related patient and public communities: Our dissemination plan for the findings in this study is twofold. For clinicians and professional societies, we will use formalized platforms of scientific communication including sharing the publication notice of the journal article and promoting it through social media. We will pursue prompt internal communication of the findings within the Veterans Affairs Health System. The findings will be shared at national and international forums including specialty conferences. For the general public, we will pursue lay media engagement through press releases of the findings and engagement with regulatory authorities.

Provenance and peer review: Not commissioned; externally peer reviewed.
