# Supplementary material for: Racial bias and reproducibility in pulse oximetry among medical and surgical inpatients in general care in the Veterans Health Administration 2013-19: multicenter, retrospective cohort study
Source: BMJ. 2022 Jul 6;378:e069775. doi: 10.1136/bmj-2021-069775 (PMC9254870; doi:10.1136/bmj-2021-069775)

Article Title
Racial Bias and Reproducibility in Pulse Oximetry Among General Care Medical and Surgical Inpatients in the Veterans Health Administration 2013-2019: A Cohort Study

Authors
Valeria S. M. Valbuena, MD, MSc*[1,2,3]; Sarah Seelye, PhD*[2]; Michael W. Sjoding, MD, MSc[4]; Thomas S. Valley, MD, MSc[2,4]; Robert P. Dickson, MD[4]; Steven E. Gay MD[4]; Dru Claar, MD[4]; Hallie C. Prescott, MD, MSc[2,4]; Theodore J. Iwashyna, MD, PhD[2,3,4]

* These authors contributed equally to this work

Affiliations
[1] Department of Surgery, University of Michigan, Ann Arbor, Michigan
[2] Veterans Affairs Center for Clinical Management Research, Ann Arbor, Michigan
[3] National Clinician Scholars Program, University of Michigan, Ann Arbor, Michigan
[4] Department of Internal Medicine, University of Michigan, Ann Arbor, Michigan

Contents
Supplemental analyses, tables, figures, and statistical code

Corresponding Author
Theodore J. Iwashyna, MD, PhD
VA CCMR
2800 Plymouth Road, NCRC Building 16, 3rd Floor
Ann Arbor, Michigan, 48104, USA
Email: tiwashyn@umich.edu
Twitter: @iwashyna

Table of Contents

Appendix 1. Comorbidities for patients with pairs of pulse oximetry and arterial oxygen saturation

Appendix 2. Analytics code and log files for results

Appendix 3. Number of paired SpO_2-SaO_2 measurements included in the study, stratified by race of Veterans and SpO_2 level and accompanying histograms of SpO_2 & SaO_2

Appendix 4. Characteristics for total VAPD hospitalizations and those with available SpO_2-SaO_2 pairs only

Appendix 5. Bland-Altman Plots and calibration plots by race and ethnicity

Appendix 6. Probability of occult hypoxemia for White and Hispanic patients

Appendix 7. Probability of occult hypoxemia for Black and White patients. All SpO_2 and SaO_2 values included

Appendix 8. Probability of occult hypoxemia for White and Black patients including a hospital-level random effect.

Appendix 9. Post hoc sensitivity analyses

Appendix 1. Comorbidities for patients with pairs of pulse oximetry and arterial oxygen saturation

	Patients with 1 pair/day	Patients with 2 pairs/day	*p*
Total pairs, N	27,023	3,016	
SpO_2 median (IQR)	96 (93, 98)	95 (92, 98)	<0.001
SaO_2, median (IQR)	94 (89.7, 96.9)	94 (89.8, 97.1)	0.186
Total patients, N	27,023	1,508	
Comorbidities, N (%)			
Congestive heart failure	9,479 (35.1)	513 (34.0)	0.401
Neurologic disease	3,328 (12.3)	179 (11.9)	0.608
Chronic pulmonary disease	14,290 (52.9)	758 (50.3)	0.048
Liver Disease	2,515 (9.3)	138 (9.2)	0.839
Diabetes without complication	6,882 (25.5)	397 (26.3)	0.456
Diabetes with complication	4,755 (17.6)	255 (16.9)	0.495
Non-metastatic cancer	2,867 (10.6)	166 (11.0)	0.625
Metastatic cancer	1,017 (3.8)	69 (4.6)	0.109
Renal disease	7,035 (26.0)	351 (23.3)	0.017
Cardiac arrhythmia	9,267 (34.3)	582 (38.6)	0.001
Valvular disease	2,029 (7.5)	134 (8.9)	0.049
Pulmonary circulation disorders	2,776 (10.3)	149 (9.9)	0.625
Peripheral vascular disorders	3,169 (11.7)	198 (13.1)	0.100
Paralysis	560 (2.1)	36 (2.4)	0.405
Hypertension with and with complication	18,769 (69.5)	1,030 (68.3)	0.344
Hypothyroidism	2,699 (10.0)	150 (10.0)	0.959
Peptic ulcer disease excluding bleeding	215 (0.8)	8 (0.5)	0.255
Lymphoma	420 (1.6)	27 (1.8)	0.472
Coagulopathy	1,722 (6.4)	112 (7.4)	0.104
Rheumatoid arthritis	535 (2.0)	23 (1.5)	0.215
Fluid and electrolyte disorders	10,042 (37.2)	543 (36.0)	0.367
Blood loss anemia	227 (0.8)	13 (0.9)	0.927
Deficiency anemia	1,969 (7.3)	91 (6.0)	0.068
Obesity	3,386 (12.5)	189 (12.5)	0.997
Weight loss	2,479 (9.2)	126 (8.4)	0.283
Alcohol abuse	2,838 (10.5)	154 (10.2)	0.721
Drug abuse	1,510 (5.6)	86 (5.7)	0.850
Depression	5,061 (18.7)	249 (16.5)	0.031
Psychosis	1,077 (4.0)	60 (4.0)	0.990

Appendix 2. Analytic code (see Supplemental file)

The stata .do files and a .log file for the results section are uploaded as additional text files.

All executable files are available in GitHub https://github.com/CCMRcodes/RacialBias

Appendix 3. Number of paired SpO_2-SaO_2 measurements included in the study, stratified by race of Veterans and SpO_2 level and accompanying histograms of SpO_2 & SaO_2

**SpO_2 level**	**89**	**90**	**91**	**92**	**93**	**94**	**95**	**96**	**97**	**98**	**99**	**100**
White	388	888	963	1619	1806	2303	2535	2571	2212	2035	1311	1765
Black	66	149	171	283	321	449	531	673	743	896	731	1225
Hispanic	24	35	35	73	88	114	138	185	166	159	165	378

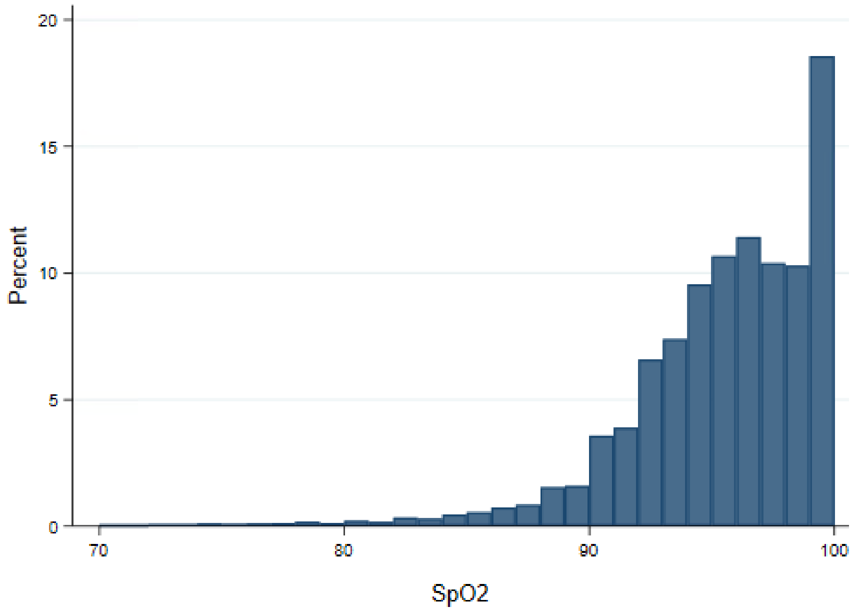

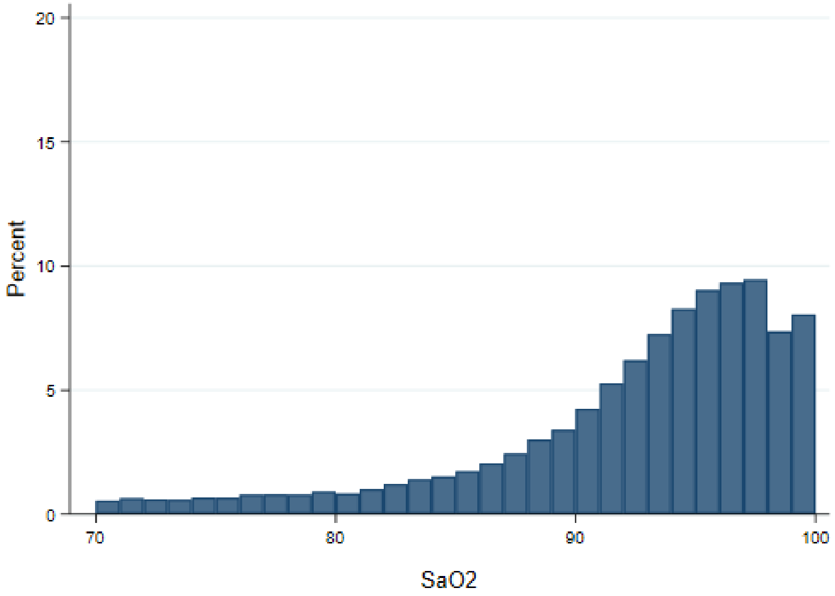

Appendix 4. Characteristics for total VAPD hospitalizations and those with available SpO_2-SaO_2 pairs only

	SpO_2-SaO_2 Pairs	VAPD
Total hospitalizations, N	27,427	3,644,559
Patient characteristics		
Age in years, median (IQR)	69 (63, 76)	67 (60, 74)
Male sex, N (%)	26,362 (96.1)	3,445,716 (94.5)
Race, N (%)		
Non-Hispanic White	20,134 (73.4)	2,607,137 (71.5)
Non-Hispanic Black	5,930 (21.6)	807,787 (22.2)
Hispanic/Latino	1,363 (5.0)	229,635 (6.3)
Primary Diagnoses, N (%)		
Chronic obstructive pulmonary disease	3,674 (13.4)	107,658 (3.0)
Respiratory failure	3,380 (12.3)	61,900 (1.7)
Septicemia	2,109 (7.7)	118,539 (3.3)
Pneumonia	1,914 (7.0)	95,402 (2.6)
Congestive heart failure	1,822 (6.6)	137,750 (3.8)
Coronary atherosclerosis	597 (2.2)	125,799 (3.5)
Diabetes with complication	536 (2.0)	69,488 (1.9)
Cardiac dysrhythmia	460 (1.7)	128,321 (3.5)
Renal failure	444 (1.6)	59,778 (1.6)
Acute myocardial infarction	328 (1.2)	50,356 (1.4)
Other	12,163 (44.4)	2,689,568 (73.8)
Comorbidities*, N (%)		
Congestive heart failure	9,620 (35.1)	752,698 (20.7)
Neurologic disease	3,350 (12.2)	277,895 (7.6)
Chronic pulmonary disease	14,569 (53.1)	926,811 (25.4)
Liver Disease	2,548 (9.3)	318,868 (8.8)
Diabetes without complication	7,020 (25.6)	851,028 (23.4)

Diabetes with complication	4,843 (17.7)	494,273 (13.4)
Non-metastatic cancer	2,870 (10.5)	396,882 (10.9)
Metastatic cancer	1,050 (3.8)	133,121 (3.7)
Renal disease	7,128 (26.0)	749,215 (20.6)
Length of hospitalization in days, median (IQR)	6 (3, 10)	4 (2, 6)
Mortality		
In-hospital mortality, N (%)	1,390 (5.1)	55,013 (1.5)
30-d mortality, N (%)	2,764 (10.1)	152,051 (4.2)

Appendix 5. Bland-Altman plots and calibration plots by race and ethnicity

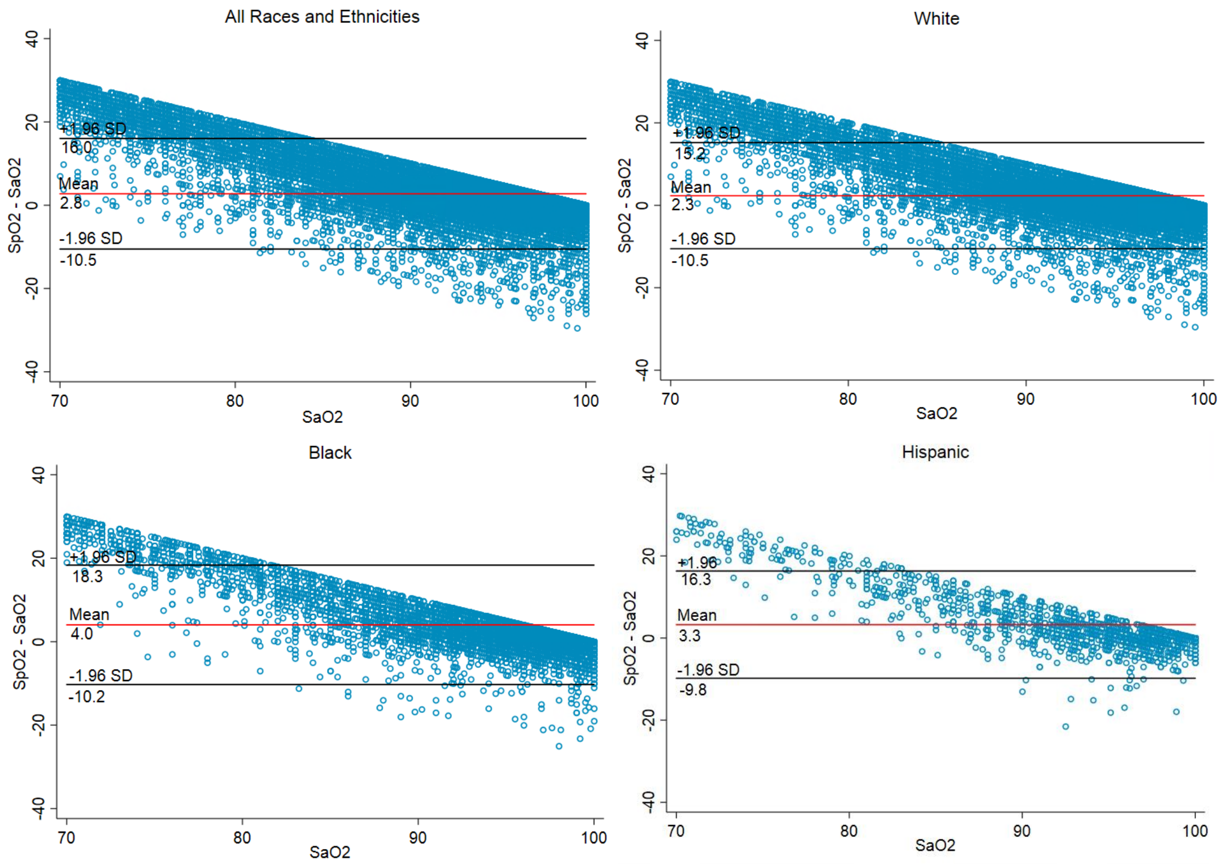

All Available SpO_2-SaO_2 Pairs				
	All (N=30,039)	White (n=21,918)	Black (n=6,498)	Hispanic (n=1,623)
Bias, %	2.8	2.3	4.0	3.3
Precision, SD	6.8	6.6	7.3	6.7
Upper limit of agreement, %	16.0	15.2	18.3	16.3
Lower limit of agreement, %	-10.5	-10.5	-10.3	-9.8
Root mean square error, %	7.3	7.0	8.3	7.4

Calibration plots by race and ethnicity

All Races

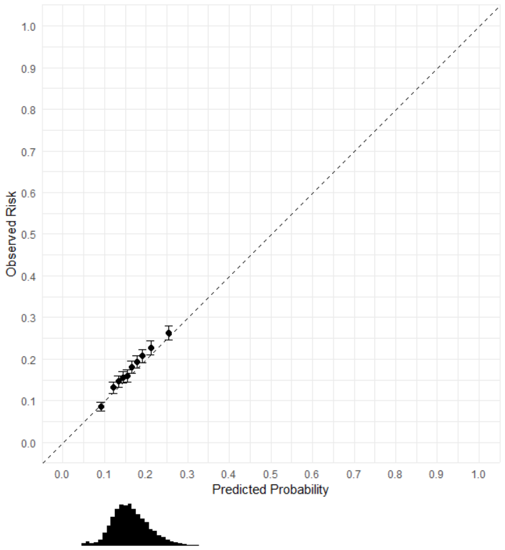

Black

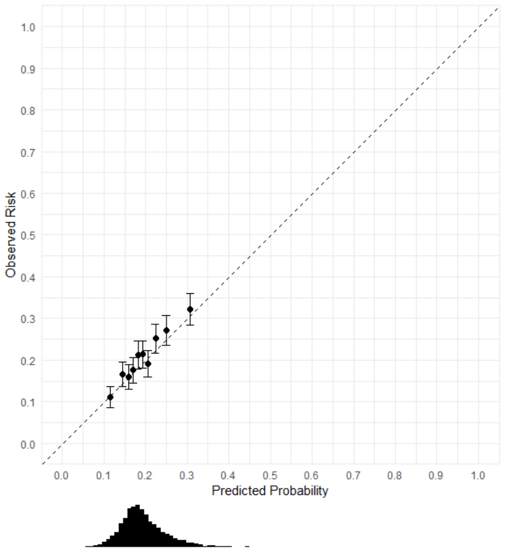

White

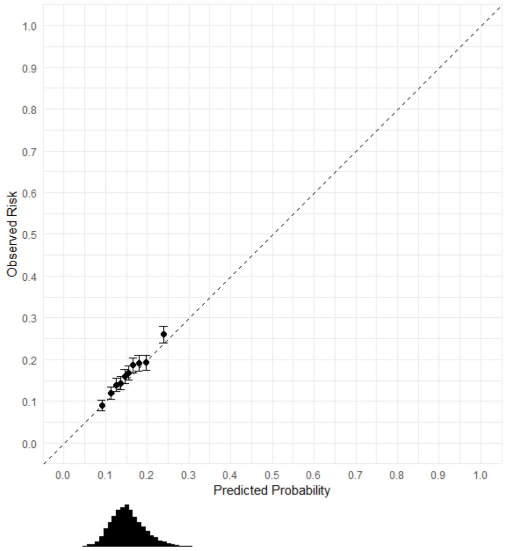

Hispanic

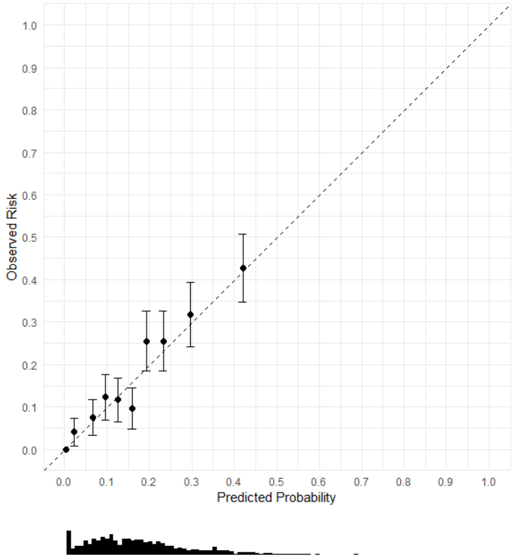

Appendix 6. Probability of occult hypoxemia for White and Hispanic patients

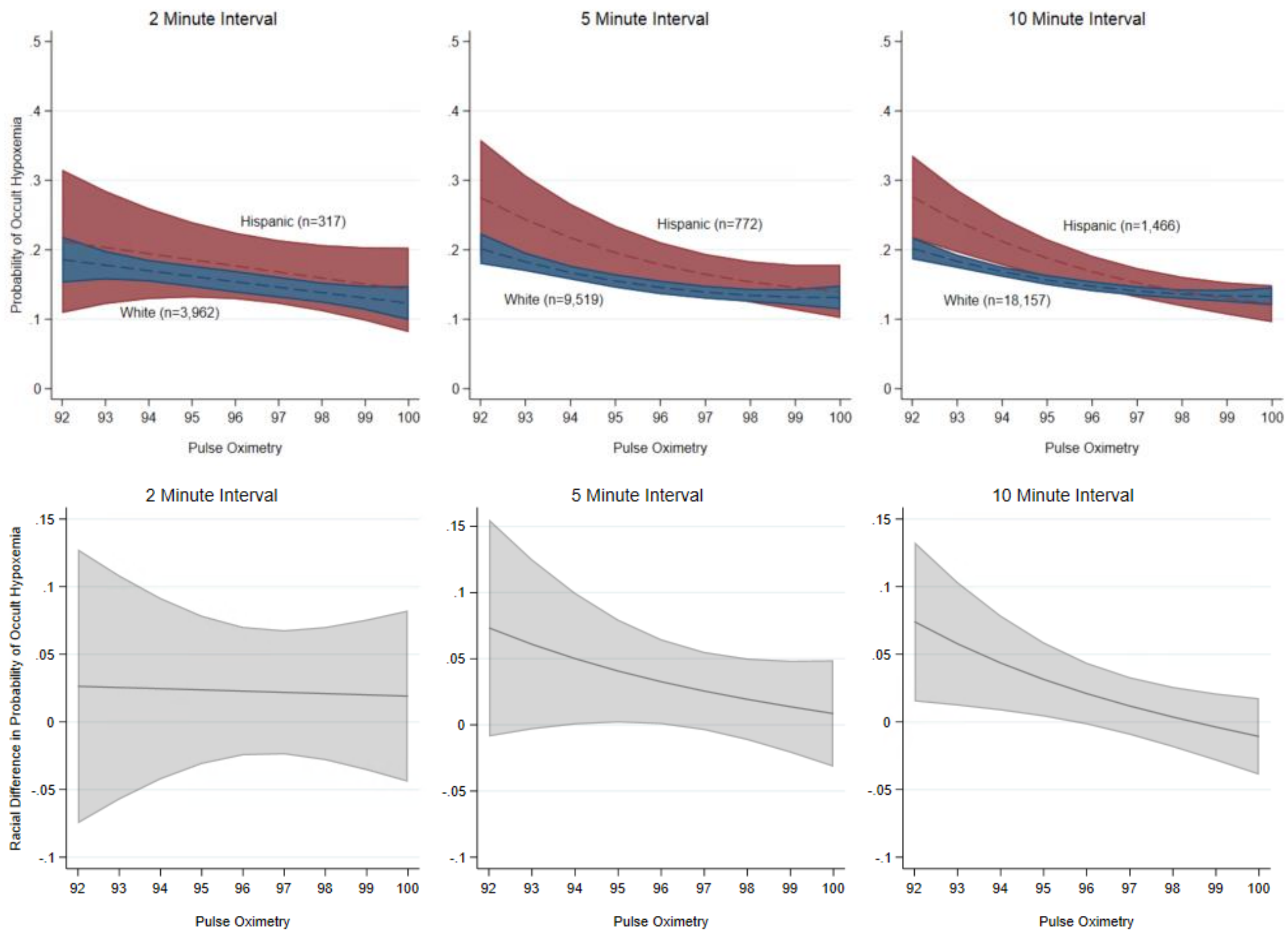

Appendix 7. Probability of occult hypoxemia for Black and White patients. All SpO_2 values included (i.e. not restricting model to $SpO_2 \leq 92\%$)

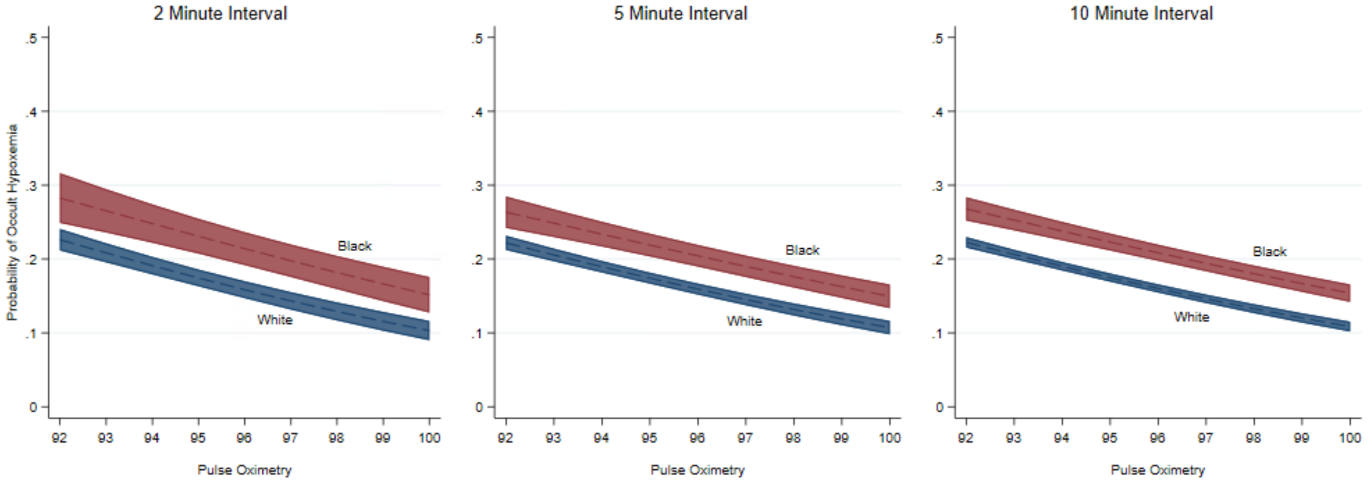

Note: When we include **all** SpO_2 values, in the 10-minute interval, 22.8% of Black patients and 18.1% of White patients had SaO_2<88%. This is a 4.6% difference (CI=3.3%, 6.0%).

Probability of occult hypoxemia for Black and White patients. All SpO_2 and SaO_2 values included (i.e. not dropping SpO_2 or SaO_2 < 70%)

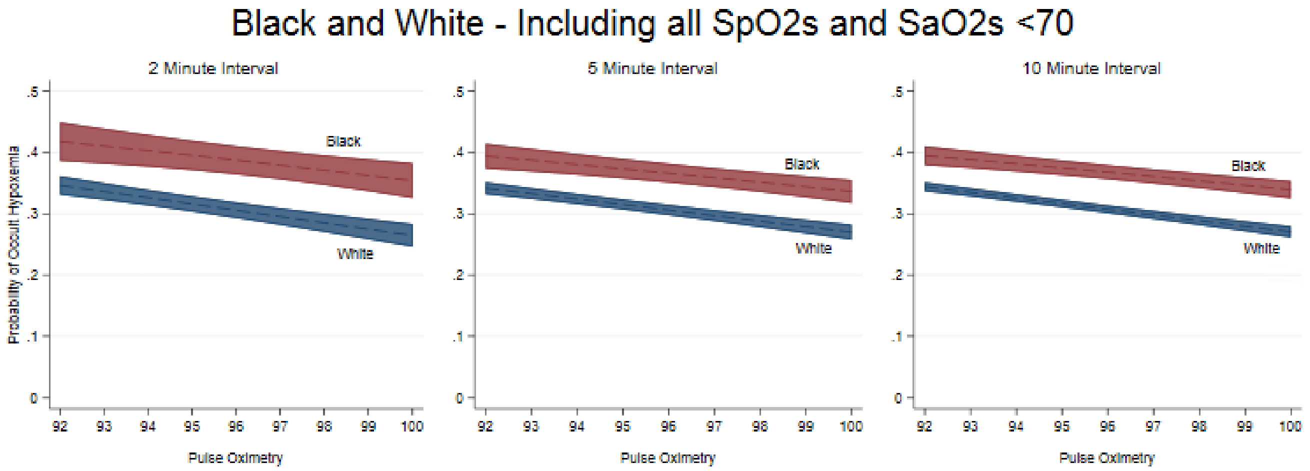

Appendix 8. Probability of occult hypoxemia for White and Black patients including a hospital-level random effect.

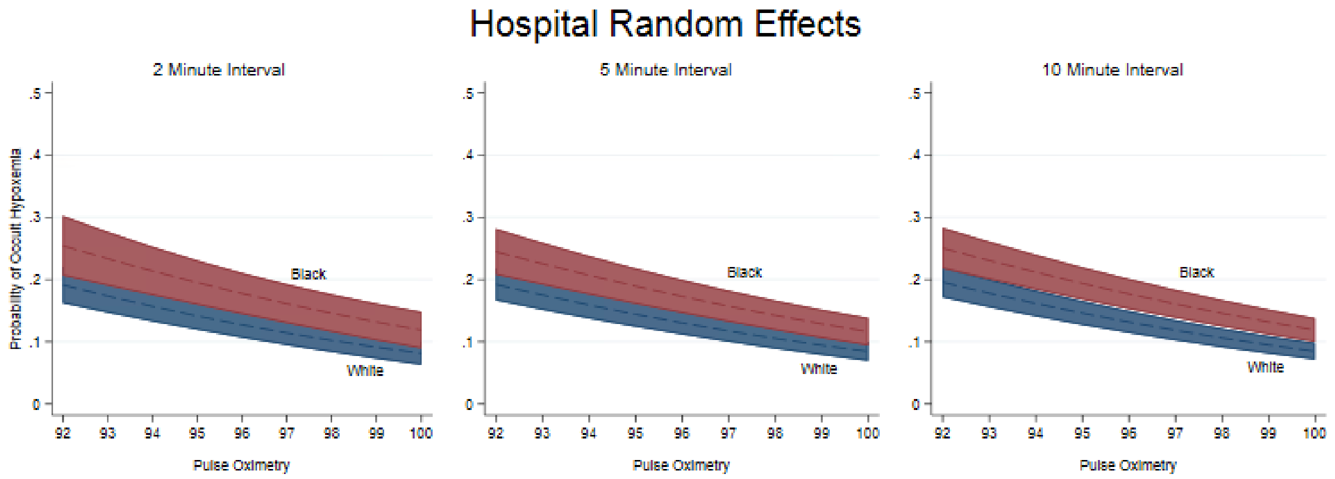

Appendix 9. Post Hoc analysis of surgical vs non-surgical patients

Of the SaO_2-SpO_2 pairs, only 580 pairs—approximately 10%—from Black patients and 1,758 pairs from White patients occur on surgical services on patient-days outside the ICU.

In the interacted analysis, there was no large difference between surgical and non-surgical patients:

> Non-surgical patients: Black patients have a 4.0% greater chance of hypoxemia than White patients (p<0.001, CI 2.7%, 5.4%)
> Surgical patients: Blacks have a 2.7% greater chance of hypoxemia than Whites (p<0.001, CI= 1.7%, 3.6%)

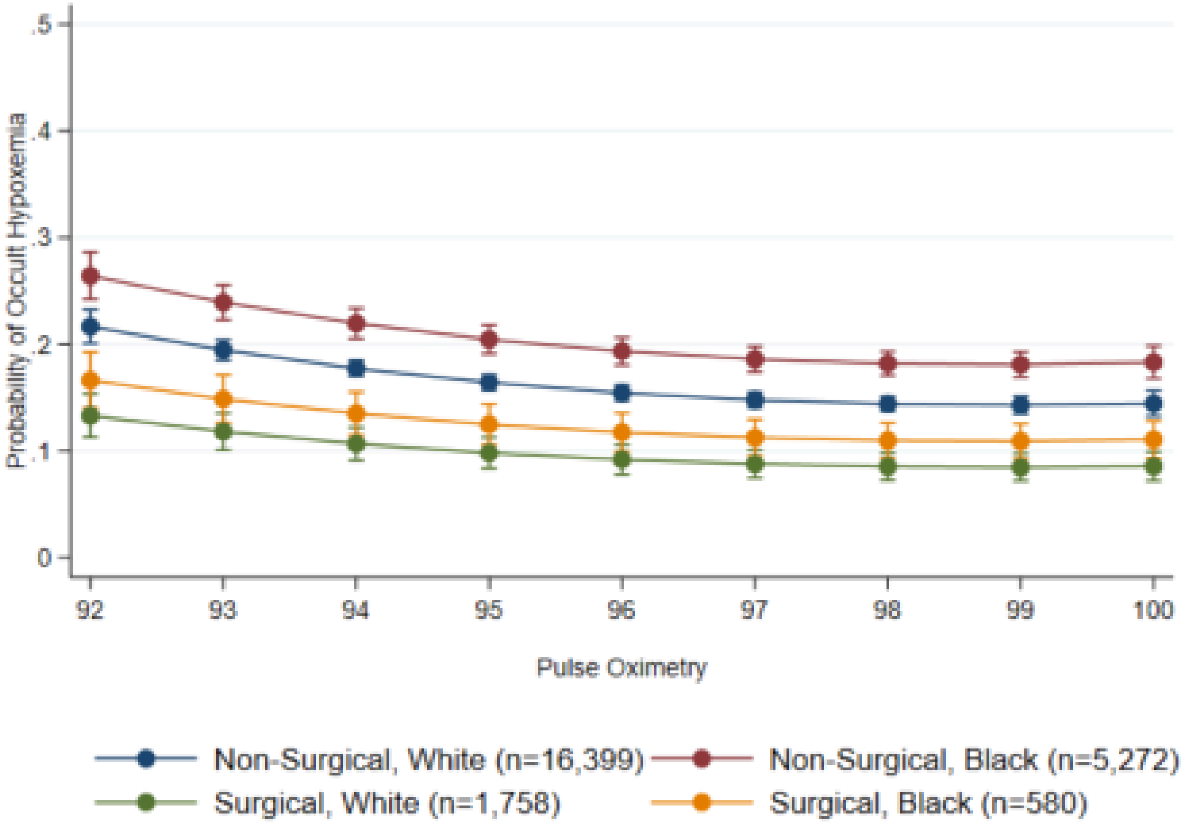

However, in fully stratified analyses in this small, post-hoc population, it appears there is no difference in occult hypoxemia by race—the point estimates and confidence intervals overlap:

White and Black Surgical Patients

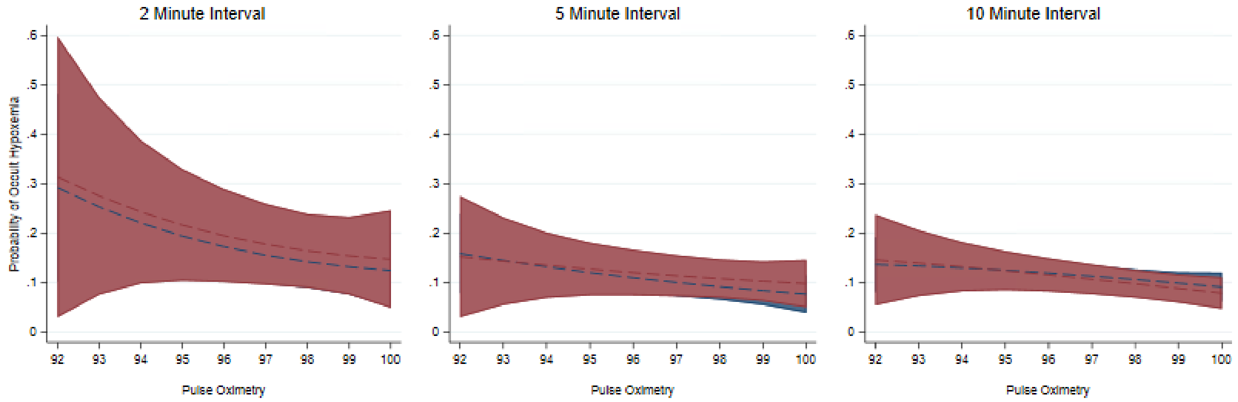

At the same subset of VA hospitals that perform surgeries, a racial differential *was* present in non-surgical patients:

White and Black Non-surgical Patients in Hospitals with Surgeries

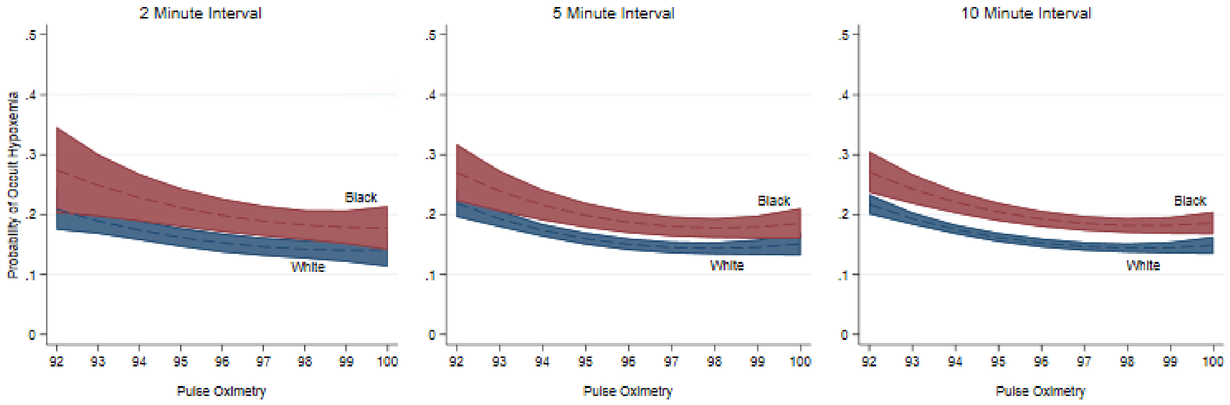

Supplement: Supplementary file 1 — Web appendix: Supplementary material [file valv069775.ww1.pdf]
